# Feature-Based Complexity Measure for Multinomial Classification Datasets

**DOI:** 10.3390/e25071000

**Published:** 2023-06-29

**Authors:** Kyle Erwin, Andries Engelbrecht

**Affiliations:** 1 Computer Science Division, Stellenbosh University, Stellenbosch 7600, South Africa; engel@sun.ac.za; 2 Department of Industrial Engineering, Stellenbosh University, Stellenbosch 7600, South Africa; 3 Center for Applied Mathematics and Bioinformatics, Gulf University for Science and Technology, Mubarak Al-Abdullah 32093, Kuwait

**Keywords:** multinomial classification datasets, classification problem complexity, feature-based complexity measures, synthetic datasets

## Abstract

Machine learning algorithms are frequently used for classification problems on tabular datasets. In order to make informed decisions about model selection and design, it is crucial to gain meaningful insights into the complexity of these datasets. Feature-based complexity measures are a set of complexity measures that evaluates how useful features are at discriminating instances of different classes. This paper, however, shows that existing feature-based measures are inadequate in accurately measuring the complexity of various synthetic classification datasets, particularly those with multiple classes. This paper proposes a new feature-based complexity measure called the F5 measure, which evaluates the discriminative power of features for each class by identifying long sequences of uninterrupted instances of the same class. It is shown that the F5 measure better represents the feature complexity of a dataset.

## 1. Introduction

Analyzing large datasets is crucial for deriving meaningful and actionable insights that go beyond simple correlations. With the advent of big data, datasets can contain millions of examples (rows) and features (columns) [1,2]. Data complexity analysis uses a broad category of measures that offer such insights. For example, the complexity of optimization problems can be quantified using landscape analysis which includes fitness landscape analysis (FLA) [3] and exploratory landscape analysis (ELA) [4]. Topological data analysis (TDA) measures the topological features in data, and the relationships between them, to assess complexity [5,6]. Complexity measures for regression problems include feature correlation measures, linearity measures, smoothness measures, and geometrical, topology, and density measures [7]. Lastly, complexity measures for classification problems focus on the geometrical complexity of the class boundary [8]. Complexity measures for classification problems include feature-based measures, linearity measures, neighborhood measures, network measures, dimensionality measures, and class imbalance measures [9]. Applications of these measures include data analysis, data pre-processing, understanding algorithm performance, meta-learning or automated machine learning, and selecting benchmarks that cover a variety of complexity characteristics [7,9,10,11,12,13,14].

This paper is concerned with feature-based complexity measures for classification problems. A classification problem is a type of supervised learning where the goal is to take advantage of geometric shapes in the data to separate instances of different classes. Feature-based complexity measures quantify the discriminative power of the descriptive features in a dataset—that is, how useful the features are in separating instances of different classes [9]. Classification datasets with highly discriminative features are considered simple, while datasets with features that exhibit little to no discriminative power are considered complex.

It is worth noting that various statistical approaches, including chi-squared statistics, ANOVA F-value, mutual information, lasso regression, two-sample t-test, Kruskal–Wallis test, Kolmogorov–Smirnov test, and more, are commonly used to estimate feature informative power and selection. These approaches have a different objective compared to feature-based complexity measures, which is to quantify feature informative power for feature selection, while feature-based complexity measures focus on estimating the complexity of the dataset. Despite this distinction, there is a similarity between these two types of approaches, making it worthwhile to explore the possibility of using these statistical measures as complexity measures, similar to how feature-based complexity measures have been utilized for feature selection.

Unfortunately, existing feature-based complexity measures are not designed to handle multinomial classification problems; they are designed for binary classification problems. These measures typically use the minimum and maximum feature values of each class to determine the overlapping region of the classes. The instances outside of this region are seen as instances that can be easily discriminated by the features. However, the use of the minimum and maximum values of each class for each feature presents two problems. Firstly, there is sensitivity to noise, as a single noisy instance could result in an overestimation of complexity. Secondly, the use of minimum and maximum values cannot estimate the complexity of real-world classification problems, such as cases where the instances of one class lie between those of another class. To handle multinomial classification problems, existing measures require decomposing the classification problem into multiple sub-problems using the one-versus-one (OVO) strategy. The average of these sub-problems is then taken as the complexity value. However, the use of OVO is computationally inefficient, and as shown in this paper, the use OVO does not properly capture the complexity of the classification problem..

This paper proposes the F5 measure, which is a new feature-based complexity measure that is designed to effectively handle multinomial classification problems. The measure determines the most discriminative feature by identifying the longest sequence of uninterrupted instances for each class for each feature. These sequences are considered to be discriminated by their respective features. The feature that discriminates the most instances is selected, and its sequences are removed from the dataset. The measure then proceeds to consider the remaining features. This process continues until either there are no more features to consider, or there are no more instances to be removed. The number of instances remaining in the dataset after this process relative to the original number of instances in the dataset is interpreted as the complexity of the dataset. It is shown that the proposed measure better represents intuitions about feature complexity. This work is useful as it can enhance the application of complexity measures in various domains, including those mentioned earlier.

The rest of the paper is organized as follows: Formal definitions for existing feature-base complexity measures are given in Section 2. Section 3 proposes a new feature-based complexity measure. Section 4 details the experiments used to demonstrate the difference between the proposed measure and existing measures and presents the results. Section 5 concludes the paper.

## 2. Feature Complexity

A tabular classification problem is defined by the data in a dataset where the objective is to correctly predict the class of each instance, assuming that each instance has only has a single class. Formally, a dataset, *T*, contains *n* instances in which each instance $\left(x_{j},y_{j}\right)$ is described by *m* descriptive features and a target feature in $y_{j}\in \left\{1,\ldots ,n_{c}\right\}$ that corresponds to its class. Feature-based complexity measures estimate how informative the *m* features are in discriminating among instances of different class labels [13]: that is to say, how useful the features are in separating the $n_{c}$ classes. The more instances that can be separated, the simpler the problem. Section 2.1, Section 2.2, Section 2.3, Section 2.4 and Section 2.5 describe existing feature-based complexity measures, namely F1, F1v, F2, F3 and F4, respectively. Note that lower values returned by the measures indicate the presence of one or more features that exhibit a large amount of discriminative power. Larger values, on the other hand, indicate that the descriptive features are discriminatively weak and, thus, are more complex.

### 2.1. Maximum Fisher’s Discriminant Ratio

Ho and Basu proposed the maximum Fisher’s discriminant ratio measure (F1) to capture the complexity of a dataset [8]. They argued that multi-dimensional classification problems are easy so long as there exists one discriminating feature. The F1 measure returns the Fisher statistics of the feature with the largest contribution to class discrimination [14]. In other words, the F1 measure identifies the feature with the largest discriminative power [8]. This paper takes the inverse of the original F1 formulation, so that the measure returns low values for simple classification problems and larger values for more complex classification problems [9]. The inverse of the F1 measure is

(1) $$F1=\frac{1}{1+\max_{i=1}^{m}r_{f_{i}}},$$

where $r_{f_{i}}$ is a discriminant ratio for each feature $f_{i}$. The discriminant ratio is calculated as

(2) $$r_{f_{i}}=\frac{\sum_{k=1}^{n_{c}}n_{c_{k}}{\left(\mu_{c_{k}}^{f_{i}}-\mu^{f_{i}}\right)}^{2}}{\sum_{k=1}^{n_{c}}\sum_{l=1}^{n_{c_{k}}}{\left(x_{l,i}^{j}-\mu_{c_{k}}^{f_{i}}\right)}^{2}},$$

where $n_{c_{k}}$ is the number of instances in class $c_{k}$, $\mu_{c_{k}}^{f_{i}}$ is the mean of feature $f_{i}$ across examples of class $c_{k}$, $\mu^{f_{i}}$ is the mean of the $f_{i}$ values across all the classes, and $x_{l,i}^{j}$ denotes the individual value of the feature $f_{i}$ for an example from class $c_{k}$ [8,11]. The computational cost of the F1 measure is O(m·n), and it returns values in (0,1] [9]. A hyperplane can be drawn perpendicular to this feature’s axis to separate the classes. Lorena et al. noted that if the required hyperplane is oblique to the feature axes, F1 may not be able to capture the simplicity of the classification problem [9].

### 2.2. Directional-Vector Maximum Fisher’s Discriminant Ratio

Orriols-Puig et al. proposed the directional-vector maximum Fisher’s discriminant ratio (F1v) as a complement to the F1 measure [15]. This measure searches for a vector which can separate instances after the instances have been projected into the vector [9,15].

The directional Fisher criterion [16] is defined as

(3) $$dF=\frac{d^{t}\mathrm{Bd}}{d^{t}\mathrm{Wd}},$$

where

(4) $$d=W^{-1}\left(\mu_{c_{1}}-\mu_{c_{2}}\right),$$

(5) $$B=\left(\mu_{c_{1}}-\mu_{c_{2}}\right){\left(\mu_{c_{1}}-\mu_{c_{2}}\right)}^{t},$$

and

(6) $$W=p_{c_{1}}\Sigma_{c_{1}}+p_{c_{2}}\Sigma_{c_{2}}.$$

Vector **d** is the directional vector onto which data is projected in order to maximize class separation, **B** is the between class scatter matrix, **W** is the within-class scatter matrix, $\mu_{c_{k}}$ is the centroid (mean vector) of class $c_{k}$, $W^{-1}$ is the pseudo-inverse of W, $p_{c_{k}}$ is the proportion of examples in class $c_{k}$, and $\Sigma_{c_{k}}$ is the scatter matrix of class $c_{k}$.

Formally, the F1v measure is

(7) $$F1v=\frac{1}{1+dF}$$

F1v was implemented for two-class classification problems and has a computational cost of $O(m\cdot n+m^{3})$. The measure can be extended to classification problems with more than two classes (referred to as multinomial classification problems) by decomposing the problem into sub-problems using a ovo strategy [9]. However, F1v for multinomial classification problems is computationally expensive with a cost of $O(m\cdot n\cdot n_{c}+m^{3}\cdot n_{c}^{2})$—assuming that each class has the same number of instances [9]. As for the F1 measure, F1v values are bounded in (0,1].

### 2.3. Volume of Overlapping Region

F2 measures the volume of the overlapping region for two-class classification problems. An overlapping region is a region in the dataset that contains instances of different classes. The F2 measure computes, for each feature, the ratio of the width of the overlapping region of the classes to the width of the feature [15]. The width of the feature is the difference between the maximum and minimum values of that feature. The measure then computes the product of these ratios. Formally, the F2 measure is

(8) $$F2=\prod_{i=1}^{m}\frac{\mathrm{overlap}\{f_{i}\}}{\mathrm{range}\{f_{i}\}}=\prod_{i=1}^{m}\frac{\max\{0,\min\max\left\{f_{i}\right\}\}}{\max\max\left\{f_{i}\right\}-\min\min\left\{f_{i}\right\}},$$

where

(9) $$\begin{array}{cc} \; & \min\max\left(f_{i}\right)=\min\left(\max\left(f_{i}^{c1}\right),\max\left(f_{i}^{c2}\right)\right),\\ \; & \max\min\left(f_{i}\right)=\max\left(\min\left(f_{i}^{c1}\right),\min\left(f_{i}^{c2}\right)\right),\\ \; & \max\max\left(f_{i}\right)=\max\left(\max\left(f_{i}^{c1}\right),\max\left(f_{i}^{c2}\right)\right),\\ \; & \min\min\left(f_{i}\right)=\min\left(\min\left(f_{i}^{c1}\right),\min\left(f_{i}^{c2}\right)\right), \end{array}$$

and $\max(f_{i}^{c_{k}})$ and $\min(f_{i}^{c_{k}}$) are the maximum and minimum values of each feature in a class $c_{k}\in \left\{1,2\right\}$, respectively.

F2 returns values in [0,1] and has a computational cost of O(m·n). For multinomial classification problems, the measure can also be extended using ovo, in which case the computational cost is $O(m\cdot n\cdot n_{c})$.

F2 can identify only one overlapping region per feature. Alternatively, F2 can be thought of as only being able to identify two hyperplanes that separate classes per feature, since it computes one overlapping region and considers the instances on either side of that region. Hu et al. noted that the F2 measure does not capture the simplicity of a linear oblique border since the measure assumes that the class boundaries are perpendicular to the features axes [17]. Lorena et al. noted that F2 values can become very small when the product is calculated over a large number of features [9]. Thus, a complex classification problem with many descriptive features may produce a low F2 value, giving the impression that the problem is simple. Lastly, a single noisy class instance could result in an overlapping region that is wider than necessary [9].

### 2.4. Maximum Individual Feature Efficiency

The maximum individual feature efficiency (F3) measure returns the ratio of the feature that can discriminate the largest number of instances relative to the total number of instances in the dataset [15]. This ratio, $n_{o}\left(f_{i}\right)$, is calculated using

(10) $$n_{o}\left(f_{i}\right)=\sum_{j=1}^{n}I\left(\left(x_{j,i}>\max\min\left(f_{i}\right)\right)\wedge \left(x_{j,i}<\min\max\left(f_{i}\right)\right)\right),$$

where *I* is an indicator function that returns 1 if its argument is true, otherwise 0, $\max\min(f_{i})$ and $\min\max(f_{i})$ are as defined in Equation (9), and ∧ is the logical and operator. The F3 measure is then defined as

(11) $$F3=\min_{i=1,\ldots ,m}\frac{n_{o}\left(f_{i}\right)}{n}.$$

As for the F2 measure, F3 has a computational cost of $O(m\cdot n\cdot n_{c})$, and it returns values in [0,1]. F3 also uses the minimum and maximum values of a feature for different classes in its calculation of complexity and therefore suffers from the same problems, namely only being able to identify one overlapping region per feature, unable to identify orthogonal hyperplanes that separate classes, and sensitivity to noise.

### 2.5. Collective Feature Efficiency

The F4 measure is similar to F3 but considers the collective discriminative power of all the features [15]. F4 selects the most discriminative feature according to the F3 ratio of each feature. Then, all the instances that are discriminated by this feature are removed from the dataset. The next most discriminative feature, with respect to the remaining instances, is then selected, and the instances that are discriminated are removed. This function, defined below, is repeated until all of the instances are discriminated or all the features have been analyzed:

(12) $$f_{\min}\left(T_{r}\right)={\left\{f_{i}|\min_{i=1,\ldots ,m}\left(n_{o}\left(f_{i}\right)\right)\right\}}_{T_{r}},$$

where $n_{o}\left(f_{i}\right)$ is computed according to Equation (10); $T_{r}$ is the dataset at round *r*, and it is defined as

(13) $$\begin{array}{lll} T_{0} & = & T\\ T_{r} & = & T_{r-1}-\left\{x_{j}|\left(x_{j,i}<\max\min\left(f_{\min}\left(T_{r-1}\right)\right)\right)\vee \left(x_{j,i}<\min\max\left(f_{\min}\left(T_{r-1}\right)\right)\right)\right\}, \end{array}$$

where $T_{0}$ is the initial dataset, $\max\min(f_{i})$ and $\min\max(f_{i})$ are as defined in Equation (9), and ∨ is the logical OR operator.

Formally, F4 is defined as

(14) $$F4=\frac{n_{o}\left(f_{\min}\left(T_{r}\right)\right)}{n},$$

where $n_{o}\left(f_{\min}\left(T_{r}\right)\right)$ measures the number of instances in the overlapping region of feature $f_{\min}$.

The F4 measure returns values in [0,1], which can be interpreted as the proportion of instances that could be discriminated by drawing hyperplanes perpendicular to the feature axes. The computational cost of F4 is $O(m^{2}\cdot n\cdot n_{c})$. However, since F4 uses the F3 measure, F4 suffers from the same problems.

## 3. Collective Feature Efficiency for Multinomial Classification Problems

This section proposes a new feature-based complexity measure referred to as the F5 measure. The F5 measure is an extension of the F4 measure, which, like its predecessor, builds upon the F3 measure. However, while the F4 measure relies on the minimum and maximum values of class instances per feature, the F5 measure identifies the longest uninterrupted sequence of instances instead. Additionally, the F5 measure takes into account the discriminative power of each feature separately for each class. These modifications are made to handle multinomial classification problems without the need for ovo decomposition.

Section 3.1 introduces the idea of walking along a feature axis used to identify sequences of instances of the same class. The process of selecting the most discriminative feature is explained in Section 3.2. Finally, the F5 measure is proposed in Section 3.3.

### 3.1. Identifying Sequences of Instances of the Same Class

To better understand the ability of a dataset to discriminate between instances of different classes, imagine performing a random walk through the data and recording the class changes from one instance to the next. Similarly, the F5 measure employs a walking strategy along each feature axis, identifying the longest uninterrupted sequences of instances of the same class. Multiple sequences of each class may exist for each feature, and such sequences can be represented by the corresponding row IDs of the instances or the feature values at the start and end of the sequence. While traversing along a feature axis, if an instance shares a value with another instance but belongs to a different class, that sequence ends, and a new sequence starts. Longer sequences represent non-overlapping areas of the feature axis where lines may be drawn perpendicular to the feature axis to separate the classes. Conversely, shorter sequences may represent noise or reflect more challenging characteristics of the feature axis that are difficult to classify. The lengths of sequences are weighted by the total number of instances of the same class. It is important to note that having a longer sequence of instances from one class does not necessarily imply greater discriminative power, especially when other classes have significantly fewer samples. To address this issue, the lengths of the sequences are weighted according to their class distribution.

There is a special case where a feature exhibits discriminatory behavior primarily toward the ends of its feature axis. For example, a long sequence of instances exists at the beginning of the feature axis, and a similarly long sequence of the same class appears at the end of the axis. Between these two sequences are shorter sequences. It is reasonable to interpret these intermediate sequences as representing overlapping regions of multiple classes, while the sequences at the beginning and end of the feature axis are discriminated by the feature. In such a case, these sequences are concatenated and treated as a single sequence. The pseudocode for identifying these sequences is provided in Algorithm 1.    
**Algorithm 1:** Identifying sequences
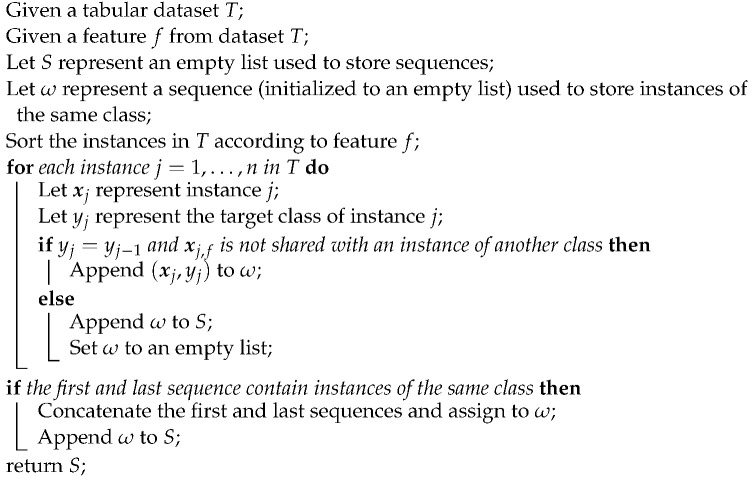


### 3.2. Selecting the Most Discriminative Feature

Algorithm 1 is used to identify sequences for each feature. Only the longest sequence of each class is considered to be discriminated by its respective feature. This approach avoids the need for a control parameter to determine the number of sequences to consider, and thus, it also avoids the need for multiple reruns of the F5 measure with differing control parameter values. The feature that discriminates the most instances is selected, and the instances that it discriminates are removed from the dataset. For example, consider a dataset with features ‘x’ and ‘y’ and classes 0 and 1. The F5 measure calculates the longest sequence of instances for class 0 and class 1 separately for features ‘x’ and ‘y’. Suppose that feature ‘x’ discriminates the greatest number of instances. These instances are removed from the dataset and the remaining feature, ‘y’, is then taken into consideration for further analysis.

### 3.3. F5 Measure

The F5 measure works as follows: The most discriminative feature is selected using the process defined in Section 3.2. The remaining features and instances are then considered, and the next most discriminative feature is selected. This process is repeated until there are no more features to consider or until all instances have been discriminated. Formally, this function is calculated as

(15) $$f_{\max}\left(T_{r}\right)={\left\{f_{i}|\max_{i=1,\ldots ,m}\left(n_{o}\left(f_{i}\right)\right)\right\}}_{T_{r}},$$

where $n_{o}\left(f_{i}\left(T_{r}\right)\right)$ returns the number of the instances in $T_{r}$ that can be discriminated by feature $f_{i}$. Dataset $T_{r}$ is the dataset of the *r*-th round after the instances from r−1 previous rounds have been removed; $T_{0}=T$. Note that when the relative entropy is calculated, the number of instances of each class is taken from $T_{0}$ and not subsequent rounds.

The F5 measure is then defined as

(16) $$F5=1-\frac{n_{o}\left(f_{\max}\left(T_{r}\right)\right)}{n},$$

where *n* is the total number of instances in *T*. The computational cost of the F5 measure is $O(m^{2}\cdot n)$, which is less than the computational cost of the F4 measure without the use of OVO. The F5 measure returns values in [0,1). A large F5 value indicates that a classification problem is complex, since it has descriptive features that discriminate few instances. Conversely, a small F5 value indicates that a classification problem has descriptive features that discriminate many instances and is therefore simple.

**Figure 1:**
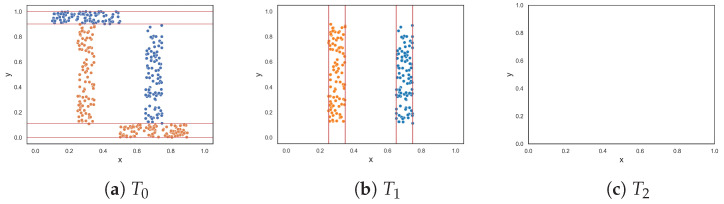
Dataset *T* at each round for the F5 measure.

To demonstrate the F5 measure, consider the synthetic dataset in Figure 1a at $T_{0}$ that contains two T-shaped data. The F5 measure examines the features and determines that the y-axis can discriminate the highest number of instances, since it has the longest uninterrupted sequences. Using these sequence, lines could be drawn perpendicular to the y-axis (i.e., near the bottom and top of the axis) to separate the instances. The resulting dataset is illustrated in Figure 1b. The F5 measure looks at the remaining feature, i.e., the x-axis. The measure finds the longest sequence of each class and removes these instances. The final dataset is shown in Figure 1c. Thus, the complexity of this dataset is 0, which makes sense.

## 4. Experiments and Analysis

This section assesses the performance of the proposed F5 measure on synthetic datasets. These datasets were crafted to contain a variety of features that are indicative of real-world classification problems. Furthermore, careful consideration was given to ensure that the datasets were easily interpretable, thus allowing the reader to form expectations about the complexity of the datasets. The datasets are grouped in the relevant sections below by problem type. Results for the proposed F5 measure and existing feature-based complexity measures are also discussed and presented.

Section 4.1 briefly details the implementation of the measures. Section 4.2 presents ten two-class classification problems. Each class contains 100 instances, and the classes are equally distributed unless stated otherwise. The features in each dataset contain continuous data in [0,1]. Likewise, Section 4.3 presents similar datasets but with three-classes.

### 4.1. Implementation

The proposed approach (F5) was implemented using Python. The source code and datasets used have been made available on GitHub (https://github.com/KyleErwin/f5-measure) (accessed on 22 June 2023). The existing feature-based complexity measures (F1 to F4 and F1v) were provided by the R package ecol (https://cran.r-project.org/web/packages/ECoL/index.html) [9] (accessed on 22 June 2023).

### 4.2. Two-Class Classification Problems

**Figure 2:**
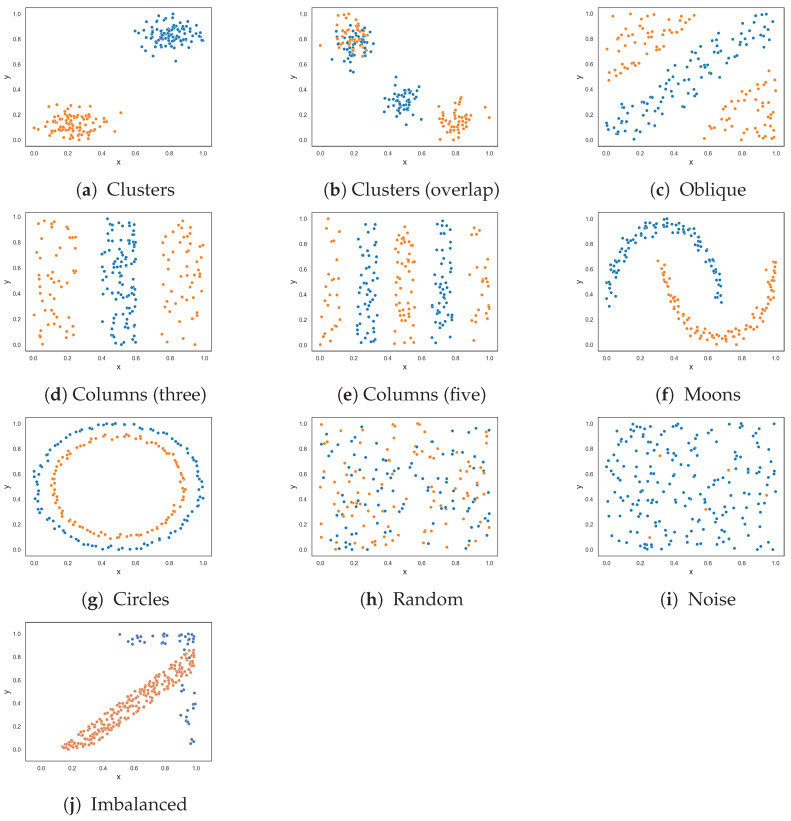
Synthetic two-class classification problems where class one is colored orange and class two is colored blue.

Figure 2 illustrates the ten two-class classification problems used to assess the performance of the feature-based complexity measures. The results are given in Table 1. Appendix A shows the resulting dataset at each round of the F5 measure for each dataset.

The clusters dataset, illustrated in Figure 2a, contains two clusters of each class. The clusters are separated by a wide margin on the y-axis. Intuitively, the dataset exhibits no complexity because of this margin. All of the feature-based complexity measures returned values near 0.0 or exactly 0.0. Thus, the measures confirmed this intuition. Figure 2b illustrates a similar, but more complex, dataset. The dataset contains two more clusters (of each class) that overlap each other. At most, 50% of the data overlaps. Thus, this problem is clearly more complex than the previous clustered dataset, and an increase in the values produced by the complexity measures was expected. Table 1 shows that values for the measures did increase. However, F1 and F3 overestimated the complexity, returning approximately 0.96 and 0.75, respectively.

The oblique dataset (Figure 2c) contains two oblique hyperplanes that separate the classes. As mentioned earlier, Lorena et al. noted the limitation of the F1 measure in capturing the simplicity of a classification problem with an oblique hyperplane [9]. This limitation extends to other measures except for the F1v measure. However, this paper offers an alternate perspective, suggesting that identifying an oblique hyperplane is not simple especially as the number of features in the dataset increases and the dataset cannot easily be visualized. All measures (including the F1v measure) returned relatively large values—thus capturing the complexity of the dataset. The F1v measure, specifically designed to detect oblique hyperplanes, returned a large value due to the presence of multiple oblique hyperplanes within the dataset and the measure being able to only identify one oblique hyperplane.

The next two datasets, illustrated in Figure 2d,e, respectively, contain columns that are separable by lines perpendicular to the x-axis. The columns alternate between instances of the two classes. The first dataset contains three columns, where one class is enclosed by the other class. The columns are clearly separable by straight lines; therefore, low complexity values were expected. The proposed F5 measure was the only measure that returned 0.0. At the opposite end, F1 and F1v produced values that were closer to 1.0. Likewise, F3 and F4 both returned 0.5. The F2 measure returned a value of 0.2. The second dataset adds two more columns (Figure 2e). Although the separability of the additional columns is obvious, the fact that more hyperplanes are required to separate the instances implies an anticipated increase in complexity. The F5 measure produces a value of 0.455, which means that 45.5% of instances are not discriminated by the features as a result of adding two more columns. The F2 returned a similar value of 0.545, while measures F3 and F4 returned even larger values of −0.75 and 0.715, respectively. The F1 and F1v measures returned values close to 1.0, suggesting that the classification problem is maximally complex.

Figure 2f,g show datasets with non-linear features. Remember that these measures quantify the discriminative power of the features rather than the linearity of the classification problem. The values returned by measures F1v, F2, F4 and F5 (i.e., between 0.1 and 0.2) indicate that the moons dataset is slightly complex. Measures F1 and F3 returned approximately 0.54 and 0.38, respectively, suggesting that the dataset is more than slightly complex. The circles dataset is a complex dataset, since the instances of one class are completely surrounded by the instances of another class. Both F1 and F1v produced a maximum value of 1.0, indicating that the problem is extremely complex, or in other words that the features cannot be used to discriminate between the classes. The F5 measure returned 0.385, while measures F2 and F4 returned values around 0.63, and the F3 measure returned 0.8. Measures F2, F3, F4 and F5 disagree about how complex the problem is. Lower values such as those of F2, F4 and F5 make sense, since instances of class two can still be separated by drawing lines between the margins of the circles. Moreover, once instances of class two are removed either by drawing lines along the x-axis or y-axis between the margins of the classes, it allows for instances of class one to be discriminated in the next round—as in the case of the F4 and F5 measures.

The next dataset, illustrated in Figure 2h, contains random data. Here, the features have very little or no discriminative power. Thus, the complexity of the dataset is at a maximum. All measures produced a value of 1.0 or near 1.0. This is good, because it means that the measures were able to capture complexity when complexity is intuitively at a maximum. Figure 2i illustrates another random dataset where class one only has five instances. These five instances might as well be considered noise, since 97.5% of the data are instances of class two. Intuitively, the complexity of this dataset is low, despite the noise. The F1 value indicates that the problem is maximally complex. Likewise, the F1v value suggests that the complexity of the problem is quite high. The F3 measure returned 0.6, and measures F2, F4 and F5 returned similar values of around 0.40. This dataset shows that a small amount of noise can affect the values produced by feature-based complexity measures.

Figure 2j shows an imbalanced dataset where class one has 250 instances and class two has 50 instances. This dataset showcases the usefulness of using the weighted lengths of sequences instead of the actual lengths. The measures F1, F1v, F2, F3, and F4 returned values ranging from 0.34583 to 0.81. However, there is only a small overlap between the two classes. The proposed F5 measure determines that the y-axis is the axis with the highest discriminative power since it discriminates 50% of class two despite being able to discriminate a larger number of class one instances on the x-axis. As a result, the F5 measure returned a score of 0.153. If the F5 measure did not weight the lengths of sequences by the class distribution, it would return a value of 0.45, thereby overestimating the complexity of the dataset.

### 4.3. Three-Class Classification Problems

**Figure 3:**
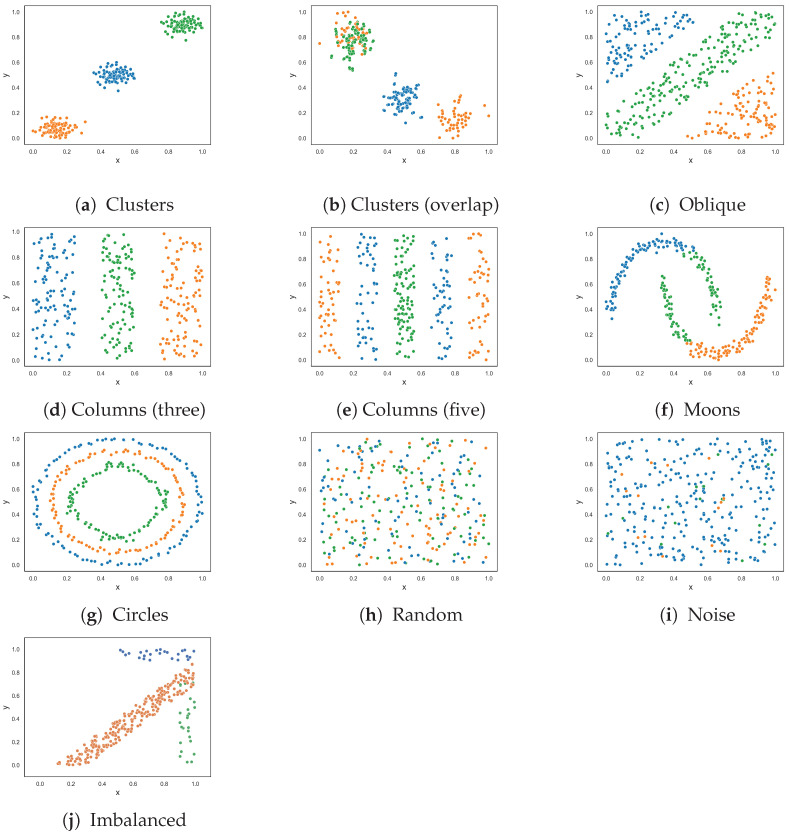
Synthetic three-class classification problem where class one is colored orange, class two is colored blue and class three is colored green.

This section follows the same experimental setup as the previous section, except that the datasets now contain three classes. Figure 3 visualizes the classification problems. The results, given in Table 2, are largely the same as in the previous section with a few exceptions. The resulting dataset at each round of the F5 measure for each three-class dataset is shown in Appendix B.

The F2 measure returned a value near 0.0 for the clusters dataset with overlapping instances, as visualized in Figure 3b. Such a value would indicate that the problem is easily solvable, but by inspection, nearly all instances of class three overlap with instances of class one.

Measures F1, F2 and F3 returned lower values than in the previous experiment for the oblique classification problem. The difference between this experiment and the last is that the existing measures make use of the ovo strategy. For example, the F1v and F4 measures return values close to 0, which suggests that the problem is similar in complexity to the clusters dataset shown in Figure 3g—which is not the case. These measures use the ovo strategy where the measures return the average complexity value of sub-problems of the dataset that only contain two classes. The F1v searches can easily identify the oblique hyperplane in each of these sub-problems. Likewise, the F4 measure determines that the collective feature efficacy of the sub-problems is very high and returns a low complexity value overall. This is an example where using the OVO strategy results in a misrepresentation about the complexity of a dataset. In contrast, the proposed F5 measure returned a value of 0.865.

All measures returned a value of 0.0 or near 0.0 for the three-columns dataset in Figure 3d except for the F1 measure. The existing feature-based complexity measures struggled with the three-class five-column dataset in Figure 3e, despite some measures having taken advantage of the OVO strategy. On the other hand, the proposed F5 measure returned a value of 0.0—which makes sense, since the x-axis is highly discriminatory amongst the classes.

The classes in the moons classification problem (Figure 3f) can be separated either by drawing lines perpendicular or orthogonal to the feature axes. The F5 measure (perpendicular lines) returned a value of 0.0, and the F1v measure (orthogonal lines) returned a value near 0.0. The F4 measure also returned a value of 0.0. The remaining measures returned low values relative to their results for the moons problem in the previous section.

The three-class circles dataset in Figure 3g adds an additional smaller circle to its two-class counterpart in Figure 2g. The additional circle makes the problem more complex and, thus, an increase in complexity values was expected. Both the F1 and F1v measures returned 1.0, which is similar to the previous experiment. The proposed F5 measure returned 0.64, which is a 0.51 increase from the previous experiment. The remaining measures, F2, F3, and F4, returned lower values compared to their values for the two-class circles problem in the previous experiment. The lower values were a result of the OVO strategy, where the measures could abuse the margin between the most outer circle and the most inner circle to discriminate instances. However, this is not how the problem would be solved in the real world and brings in further doubt about the usefulness of the OVO strategy.

For the random dataset in Figure 3h, the measures returned values near 1.0 or 1.0, which is expected. The imbalanced random dataset (Figure 3i) contains five instances of class one, five instances of class three, and all remaining instances are of class two. The F1 measure returned a value near 1.0, indicating that the dataset is as complex as the previous dataset, which is not the case. The values obtained from measures F2, F3, F4, and F5 are similar to each other and indicate a decrease in complexity compared to the previous dataset.

The imbalanced dataset in Figure 3j has 250 instances of class one, 25 instances of class two, and 25 instances of class three. Similar to the two-class imbalanced dataset, the F5 measure selects the y-axis as the most discriminative feature since it discriminates 100% of class two. However, unlike the two-class imbalanced dataset, the measures F1v, F2, F3, and F4 returned values near 0.0 due to the use of the OVO strategy. The F5 measure returned 0.14.

## 5. Conclusions

Meaningful insights into data help researchers to understand the problem being solved. Without such insights, time and effort are wasted. Complexity measures are tools designed for deriving such insights into data. This paper focused on feature-based complexity measures, which assess the discriminative power of descriptive features to separate instances of different classes within a dataset. The findings of this research indicate that current feature-based complexity measures generally do not perform well when applied to multinomial classification problems.

This paper proposed a new feature-based complexity measure, the F5 measure. This measure identifies uninterrupted sequences of instances belonging to the same class for each feature. The sequences correspond to instances that are discriminated by the features. The feature that discriminates the highest number of instances is identified as the most discriminant feature. Instances discriminated by this feature are removed and the feature is no longer considered. This process repeats until all instances have been removed or there are no more features to consider. The complexity score is the proportion of instances remaining in the dataset relative to the total number of instances in the dataset. The proposed measure is shown to accurately capture the feature complexity on a variety of synthetic datasets better than existing measures—especially multinomial datasets.

The work in this paper can be continued in the following ways (but it is not limited to them): Feature-based complexity measures have previously been used as feature selection tools, since the measures identify the most discriminative features in a dataset. Thus, an idea for future work is to investigate the performance of the F5 measure as a means of feature selection and to compare it with other feature-based complexity measures and existing feature-selection strategies. Similarly, exploring the use of commonly employed statistics used for feature selection (such as chi-squared statistics, ANOVA F-value, mutual information, lasso regression, two-sample t-test, Kruskal–Wallis test, Kolmogorov–Smirnov test and more) as complexity measures would provide valuable insights and an alternative perspective on feature-based complexity. Another idea for future work is to use the F5 measure as a meta-characteristic in automated machine learning and to investigate whether it leads to better performance. Additionally, the introduction of a hyperparameter to set a minimum sequence length would allow researchers to systematically explore the impact of different minimum lengths on the complexity measure, providing lower and upper bounds for dataset complexity. The number of removals required in the F5 measure grows as the number of classes increases, which may become costly or lead to an inaccurate assessment of complexity. To address the issue, the same feature could be selected multiple times in proportion to the number of classes. Future work should also include comparisons with performance measures obtained from machine learning algorithms on synthetic and real-world datasets to gain a better understanding of the relationship between complexity and predictive performance.

## Figures and Tables

**Table 1:** Feature-based complexity results for each synthetic two-class classification problem.

Dataset	F1	F1v	F2	F3	F4	F5
Clusters	0.06083	0.00646	0.0	0.0	0.0	0.0
Clusters (overlap)	0.95804	0.41352	0.42286	0.745	0.435	0.42
Oblique	0.99513	0.96112	0.97998	0.98	0.975	0.865
Columns (three)	0.9996	0.99677	0.1967	0.5	0.5	0.0
Columns (five)	0.99942	0.99537	0.54475	0.75	0.715	0.455
Moons	0.5366	0.11538	0.13598	0.38	0.195	0.185
Circles	1.0	0.99999	0.65573	0.8	0.61	0.385
Random	0.99969	0.99769	0.96479	0.965	0.945	0.905
Noise	0.99803	0.86552	0.44912	0.6	0.41	0.4
Imbalanced	0.81469	0.34583	0.45127	0.653	0.543	0.153

**Table 2:** Feature-based complexity results for each synthetic three-class classification problem.

Dataset	F1	F1v	F2	F3	F4	F5
Clusters	0.02158	0.00483	0.0	0.0	0.0	0.0
Clusters (overlap)	0.60384	0.20362	0.06118	0.40906	0.39727	0.407
Oblique	0.49138	0.02978	0.18377	0.44333	0.07111	0.865
Columns (three)	0.51683	0.01993	0.0	0.0	0.0	0.0
Columns (five)	0.99918	0.99518	0.29072	0.5	0.5	0.0
Moons	0.33933	0.04677	0.12085	0.28502	0.0	0.0
Circles	1.0	1.0	0.53147	0.76167	0.56	0.65
Random	0.99469	0.97009	0.93678	0.96124	0.93764	0.91
Noise	0.99434	0.82305	0.61968	0.7459	0.65457	0.72
Imbalanced	0.74088	0.04346	0.02574	0.06303	0.01697	0.14

## Data Availability

Not applicable.

## Abbreviations

The following abbreviations are used in this manuscript:

ELA	Exploratory Landscape Analysis
FLA	Fitness Landscape Analysis
OVO	One-versus-One
TDA	Topological Data Analysis
